# Sparsentan for the Treatment of Immunoglobulin A Nephropathy: An Innovative Concept for Economic Modelling

**DOI:** 10.3390/jcm15114201

**Published:** 2026-05-29

**Authors:** Garth Baxter, Antonio Ramirez de Arellano, Tom Edmonds

**Affiliations:** 1 CSL Vifor, Maidenhead SL6 8AA, UK; garth.baxter@viforpharma.com; 2 CSL Vifor, 8152 Zürich, Switzerland; antonio.ramirez@viforpharma.com; 3 Initiate Consultancy, London W1B 5BG, UK

**Keywords:** economic modelling, chronic kidney disease, IgA nephropathy, nephrology, sparsentan

## Abstract

**Background/Objectives**: Immunoglobulin A nephropathy (IgAN) is a type of chronic kidney disease (CKD) and the most common cause of kidney failure in patients <40 years of age. Previous economic models in CKD have generally defined health states solely by the progression of CKD. This manuscript presents an alternative method which also considers the level of proteinuria in a CKD patient. **Methods**: A cohort-level state transition model was developed, comparing the health benefits of sparsentan, a dual endothelin angiotensin receptor antagonist, to irbesartan, an angiotensin receptor blocker, in IgAN. Within four UP/C (proteinuria) states, patients are assigned to three sub-health states according to CKD stage. Patients with end-stage renal disease are grouped together, irrespective of UP/C, and are stratified instead by renal replacement therapy modality. Transition matrices are derived from a combination of data from PROTECT, a clinical trial comparing sparsentan to irbesartan, and the UK RaDaR registry. Health-related quality of life data from a general CKD population is used as a proxy. **Results**: Patients with IgAN who were modelled to receive treatment with sparsentan had an estimated total undiscounted life years of 25.5 years, a gain of 0.9 years in comparison with irbesartan. Patients were also more likely to spend more time in earlier CKD stages while in pre-ESRD. This translated to significant quality-adjusted life year gains for patients treated with sparsentan in comparison with irbesartan. **Conclusions**: This study presents a new structure for health economic models in IgAN that more comprehensively captures the effect of proteinuria in combination with CKD progression. This new approach ultimately allows for the more robust implementation of clinical trial data in IgAN and estimates of the cost-effectiveness of new treatments.

## 1. Introduction

### 1.1. Immunoglobulin A Nephropathy

Immunoglobulin A nephropathy, or IgAN, is the most common cause of kidney failure (or chronic kidney disease, CKD) in patients <40 years of age [1]. It is an immune complex-mediated form of glomerulonephritis, a group of diseases characterised by damage to the blood vessels in the kidney, which filter waste and remove fluids from the blood (the glomeruli). IgAN specifically sees the build-up of immunoglobulin A in the glomeruli, leading to inflammation and subsequent damage of the glomerular filtration barrier [2,3].

As kidney damage accumulates, patients can initially suffer from symptoms such as hypertension and oedema. IgAN patients are very likely to experience kidney failure as their disease progresses and, as such, experience symptoms typical of kidney failure, such as fever and flank pain, as well as other symptoms such as haematuria and loin pain [1,4,5]. IgAN leads to quicker disease progression than other causes of CKD. As such, there is a shorter window in which to administer treatments, and less time before the more advanced disease stages are reached [1,6].

As with other CKD aetiologies, patients with IgAN may receive dialysis treatment and a possible kidney transplant. However, dialysis is associated with a significant impact on a patient’s livelihood, and a kidney transplant may not prevent disease recurrence [7,8]. IgAN itself has no cure, with the goal of treatment being to slow down its rapid advancement. Angiotensin-converting enzyme inhibitors and angiotensin receptor blockers, collectively referred to as renin angiotensin–angiotensin system inhibitors (RAASi), are generally the first treatment option and are used to manage blood pressure. Patients with persistent proteinuria (high levels of protein in urine) may also be prescribed immunosuppressants. In addition, SGLT2 inhibitors are becoming an increasingly common option for CKD patients who have already optimised other treatments [9].

### 1.2. Sparsentan

Sparsentan is a non-immunosuppressive, single-molecule dual endothelin–angiotensin receptor antagonist. By blocking the receptors for endothelin and angiotensin, two hormones which contribute to inflammation and kidney damage, sparsentan reduces proteinuria and, therefore, slows down the progress of CKD [10]. Clinical studies in CKD patients have shown that sparsentan, after RAASi optimisation, reduced proteinuria to a greater extent than RAASi monotherapy [11,12,13].

The PROTECT study, a Phase 3, double-blind, randomised, active-controlled trial, compared the efficacy of sparsentan in reducing proteinuria in patients with biopsy-proven IgAN at high risk of disease progression versus irbesartan, an angiotensin receptor blocker. Sparsentan demonstrated a significantly greater improvement in proteinuria, as measured by the protein/creatinine ratio in urine samples (UP/C) [11].

### 1.3. Scope

Health economic modelling is a crucial part of health technology assessments (HTAs), allowing HTA bodies to make decisions regarding the allocation of healthcare resources in a cost-effective manner. Previous economic models in CKD have generally defined health states solely by the progression of CKD, mostly using estimated glomerular filtration rate (eGFR) [14,15,16,17,18,19,20,21]. This manuscript presents an alternative method of modelling CKD, which also considers the level of proteinuria in a CKD patient. It also validates the model using published real-world evidence in IgAN and quantifies the health benefits of treatment with sparsentan based on the results of the PROTECT clinical trial.

## 2. Materials and Methods

### 2.1. Patient Population

The target patient population for this analysis is aligned with the licensed patient population for sparsentan based on the results of the PROTECT study: adult patients with primary IgAN who have a urine protein excretion (UPE) of ≥1.0g/day (UP/C ≥ 0.75 g/g). Some patients experienced a decline in UPE between enrolment and treatment initiation [11]. The baseline characteristics of the modelled patient population are presented in Table 1 based on the results of the PROTECT clinical trial [11].

### 2.2. Model Structure

The model aimed to estimate the health benefits of treatment with sparsentan compared to irbesartan, the active comparator in the PROTECT trial. It is a cohort-level state transition model which utilises clinically established definitions of stages of CKD in Kidney Disease: Improving Global Outcomes (KDIGO) guidelines [22]. These stages are defined based on eGFR, a measure of how well a patient’s kidneys can filter waste, and are displayed in Table 2.

Previous economic models in IgAN, and CKD more generally, have typically modelled treatment effects based on patient progression through CKD stages defined by eGFR alone, or based on proteinuria alone [14,15,16,17,18,19,20,21]. However, both eGFR and proteinuria are independent predictors of patient prognosis. While eGFR provides a direct quantification of a patient’s kidney function, proteinuria is highly predictive of the rate of decline of kidney function [23]. Proteinuria also has the benefit of being more sensitive to changes in treatment and, consequently, is typically used as the primary endpoint of clinical trials in IgAN [24].

Therefore, the developed model considers both CKD stage (defined by eGFR) and a patient’s level of proteinuria. Data on CKD stage transitions from both the PROTECT study and the world’s largest registry of rare renal disease, UK RaDaR, were used to estimate transitions between CKD (as measured by eGFR) stages within UP/C states [6,11]. The RaDaR dataset defined subgroups of the full cohort, including a subgroup that would meet the inclusion criteria of a generic clinical trial for IgAN, defined as Population 4 in Pitcher et al. [6]. These inclusion criteria aligned with the PROTECT trial. Population 4 was then match-adjusted to align baseline characteristics to the PROTECT trial.

**Figure 1:**
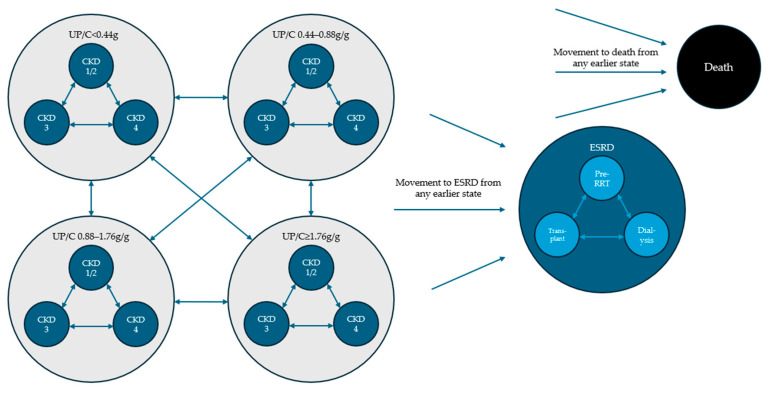
Model structure by CKD and UP/C states. Whilst not expected to be clinically plausible, backwards transitions and transitions that skipped stages (i.e., going from CKD1/2 to CKD4 or vice versa) were not strictly disallowed in the study’s model. This was to account for natural fluctuations in eGFR readings at the time of observation (for example, a patient at the boundary of two CKD states transitioning back and forth between observations) to avoid additional manipulation or added constraints to the data. Nevertheless, in the PROTECT study, there was only one instance of a patient transitioning to two states healthier (CKD5 to CKD3) out of all observed patients and cycles. Likewise, in the RaDaR dataset, there was only one observed instance of improving two states in a single transition (CKD4 to CKD1&2). Abbreviations: CKD, chronic kidney disease; ESRD, end-stage renal disease; RRT, renal replacement therapy; UP/C, urine protein/creatinine ratio.

As such, the model considers patient outcomes, including quality of life, mortality, and healthcare resource utilisation (HCRU), conditional on both CKD stage and proteinuria level, rather than just the former. As shown in Figure 1, the model utilises 15 states (excluding death): three CKD states (CKD1/2, CKD3, and CKD4) for each of the four UP/C states (<0.44 g/g, 0.44–0.88 g/g, 0.88–1.76 g/g, and ≥1.76 g/g), equating to a total of 12 states, with an additional three health states within ESRD (CKD5) for pre-renal replacement therapy (RRT), dialysis, and transplant.

UP/C states are split into four bandings, derived from the KDIGO guidelines and Pitcher et al. [6,22]. Because these transitions are different for sparsentan and irbesartan in the PROTECT trial, they are the main driver of the differences in efficacy between the two drugs. Within the UP/C states, patients are assigned to sub-health states according to CKD stage. The first two stages of CKD, CKD1 and CKD2, are considered together because of their similar HCRU and QoL profiles. Patients in CKD5 are considered to have end-stage renal disease (ESRD) and are grouped together irrespective of their UP/C, with stratifications based on RRT modality.

Figure 1 is a schematic of this model structure. As the schematic shows, patients could transition from any UP/C state to any other UP/C state and, from there, onto ESRD and then death. Within each UP/C state, patients could transition from any CKD state to any other CKD state, and within the ESRD state, patients could transition between pre-RRT, dialysis, and transplant.

The model had a lifetime horizon to reflect the lifelong, incurable nature of IgAN. The cycle length was 12 weeks, reflecting the frequency of data collection in the PROTECT trial. The treatment effect of sparsentan, characterised by control of UP/C, following the end of the trial, was assumed to continue the course of the plateaued UP/C observed in the trial period by using the same transition probabilities as used from Weeks 12 to 108.

### 2.3. Health State Transitions

Transitions between the health states defined in the model were based on three separate sources: PROTECT data for transitions between the UP/C health states and health states defined by CKD1 to CKD4 (within each UP/C state), RaDaR data for transitions from CKD 4 and to CKD5, and UK Renal Registry (UKRR) data for transitions within ESRD [6,11,25]. Patients in RaDaR were matched in terms of clinical and demographic characteristics and inclusion criteria to the PROTECT clinical trial. This approach used weights obtained from a logistic regression model (for full details on this matching, see Document S1: Logistic regression weightings).

The transition matrix for the first 12-week cycle is treatment-specific and is derived from a combination of the first 12 weeks of PROTECT (for treatment-specific UP/C transitions and treatment-agnostic early CKD stage transitions) and the RaDaR dataset (for treatment-agnostic late CKD stage transitions) [6,11]. This is because the change in UP/C observed in the first 12 weeks of PROTECT was substantial. If it were used and extrapolated over the entire stage of the model, it would result in a significant overestimation of the sparsentan treatment effect over the lifetime model horizon. From Week 12 to Week 108 (the end of the PROTECT trial period), another treatment-specific transition matrix is derived from the PROTECT and RaDaR data. A uniform transition matrix is employed from this point forward, made from extrapolated UP/C and CKD stage transitions based on the results of PROTECT and RaDaR.

Transitions between UP/C health states were based on patient-level data from PROTECT. Patients were sorted into four groups based on their UP/C at baseline, with movements between these groups at 12-week observation intervals recorded in both treatment arms and entered into the model. From Week 106 onwards, transitions were based on the uniform transition matrix described above.

Transition probabilities between CKD stages were calculated from the observational counts of patients transitioning. Patients were grouped by their time-averaged UP/C readings. However, CKD transitions were treatment agnostic (no separation of results based on treatment). Transitions originating from states CKD 1–3 were informed by PROTECT and by RaDaR for transitions originating from CKD 4–5.

In the model, patients who progress to ESRD are grouped together regardless of their UP/C and distributed across three states: those who are not receiving RRT, those receiving dialysis, and those who have received a kidney transplant. A constant transition matrix was applied for these three states, with transition probabilities taken from the NICE technology appraisal guidance for targeted-related budesonide in treating IgAN (NICE TA775) [14,26,27].

A more detailed explanation of the structure of the transition matrices can be found in the Document S2: Transition matrices calculations.

### 2.4. Adverse Events, Treatment Discontinuations, and Mortality

Data on adverse events and treatment discontinuations in the model was sourced from the PROTECT trial. All treatment-emergent adverse events (TEAE) that occurred in ≥5% of patients in either treatment arm were modelled using one-off costs and one-off utility decrements in the first cycle, sourced from Sullivan et al. 2011 [28]. The disutilities applied for adverse events are outlined in Table 3. All disutilities were set to last for 7 days.

Discontinuation of sparsentan was applied as a constant per-cycle rate based on rates of treatment discontinuation in PROTECT, resulting in an annual discontinuation probability of 7.08% and a per-cycle discontinuation probability of 1.68%. Additional discontinuations were included by assuming that treatment would be stopped if sparsentan did not result in a sufficient treatment effect.

Mortality rates could not be modelled using PROTECT data because the rate of mortality in the trial was low. A systematic literature review found no published data on IgAN-specific mortality rates [13]. Therefore, data from the 2024 KDIGO draft guidelines were used to apply mortality hazard ratios (HRs) for CKD1/2, CKD3, and CKD4 patients, as well as ESRD patients who had not undergone RRT, versus all-cause mortality in the population of England adjusted for age and sex [29]. For ESRD patients who were undergoing dialysis or who had received a kidney transplant, HRs calculated by Neovius et al. 2014 were multiplied by the CKD4 HR [30]. These HRs, as applied in the model, are shown in Table 4.

### 2.5. Health-Related Quality of Life

EQ-5D data was collected in the PROTECT trial, but this was unsuitable for use in the model because the data was not broken down sufficiently to provide inputs for all the modelled health states. Because a systematic literature review found no health-related quality of life (HRQoL) data for IgAN specifically, CKD was used as a proxy in the model; Cooper et al. 2020 was selected for use on the basis of its inclusion in previous NICE submissions for treatments in IgAN and CKD [26,31,32]. The utility values reported by Cooper et al. are shown in Table 5.

### 2.6. Model Validation

Model predictions were validated against real-world data reported by Pitcher et al. 2023, who reported ESRD-free survival for patients categorised by time-averaged UP/C level (<0.44 g/g, 0.44 to <0.88 g/g, 0.88 to <1.76 g/g, and ≥1.76 g/g) based on an analysis of long-term follow-up data collected in RaDaR [6]. The economic model was parameterised to reflect three of these groups (0.44 to <0.88 g/g, 0.88 to <1.76 g/g, and ≥1.76 g/g), reflecting those groups who would potentially be eligible for treatment with sparsentan. In these validation scenarios, patients were assumed to not transition between UP/C health states, consistent with the time-averaged approach presented by Pitcher et al. 2023 [6]. Model predictions of ESRD-free survival for irbesartan-treated patients were compared with those presented by Pitcher et al. 2023 [6]. Validation was assessed in terms of visual inspection, R^2^, and root mean square error (RMSE).

## 3. Results

### 3.1. Model Validation

**Figure 2:**
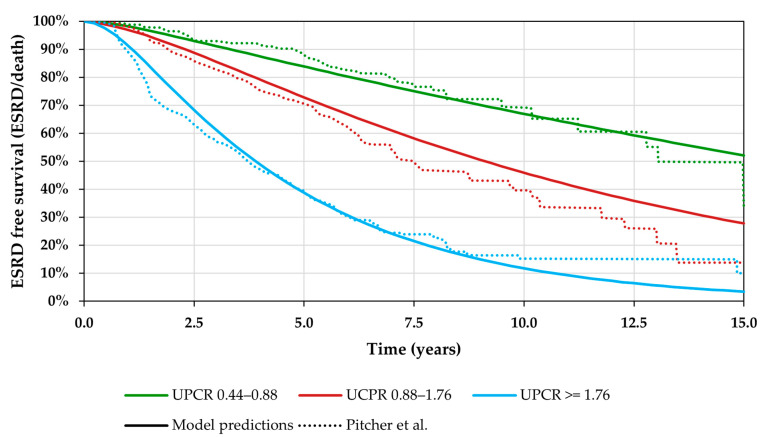
Model predictions and Kaplan–Meier estimates. Kaplan–Meier estimates based on digitisation of the data presented by Pitcher et al. Abbreviations: ESRD, end-stage renal disease; UPCR, urine protein/creatinine ratio. The solid lines refer to the model predictions, and the dotted lines to the data in Pitcher et al. [6]. The black colour de-notes that this is applicable to all three UPCR groups.

External validation exercises found that the model predictions of ESRD-free survival for patients treated with irbesartan were consistent with the findings presented by Pitcher et al. 2023 [6]. Model predictions and Kaplan–Meier estimates based on digitisation of the data presented by Pitcher et al. are presented in Figure 2. R^2^ was high, with values of 0.967, 0.993, and 0.984 in UP/C states defined by 0.44 to <0.88 g/g, 0.88 to <1.76 g/g, and ≥1.76 g/g, respectively. Similarly, RMSE over a 15-year horizon was generally low, with values of 3.7%, 7.8%, and 5.6% for UP/C states defined by 0.44 to <0.88 g/g, 0.88 to <1.76 g/g, and ≥1.76 g/g, respectively. Visual inspection showed good alignment between the model predictions and the published real-world evidence in IgAN.

### 3.2. Model Application

**Figure 3:**
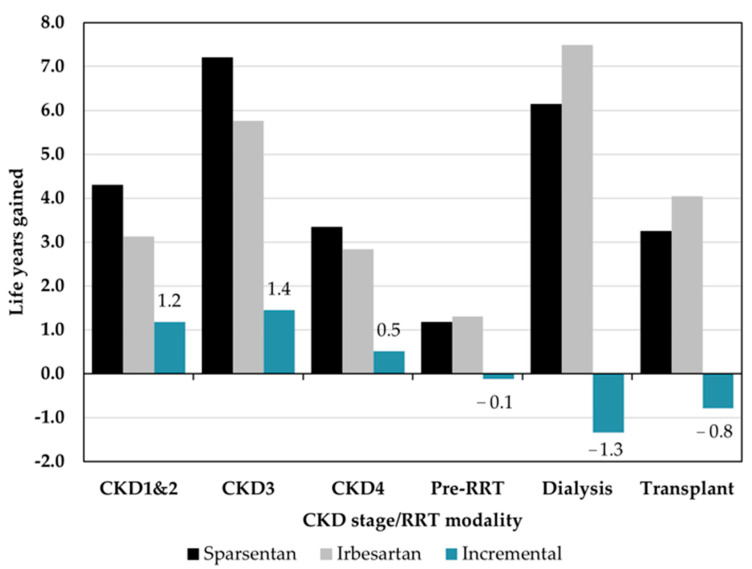
Summary of life years gained by CKD stage/RRT modality. Abbreviations: CKD, chronic kidney disease; RRT, renal replacement therapy.

Patients treated with irbesartan were estimated to gain a total of 24.6 life years over a lifetime model horizon, with an average of 12.8 years spent in the ESRD health state (Table 6). Sensitivity analysis of these results can be found in the Supplementary Materials, in the form of tornado plots (Figure S1: Life year tornado plot; Figure S2: QALY tornado plot). Life expectancy combined with disease progression and treatment modality results in total quality-adjusted life year (QALY) gains of 15.7 QALYs undiscounted or 10.7 QALYs when discounted at 3.5% per annum. Treatment with sparsentan was estimated to delay disease progression, consistent with the results of the PROTECT clinical trial, leading to improved patient outcomes. Patients with IgAN who were modelled to receive treatment with sparsentan had estimated total undiscounted life years of 25.5 years, a gain of 0.9 years in comparison with irbesartan-treated patients. Patients were also more likely to spend more time in earlier CKD stages while pre-ESRD, with an additional 1.2 years in CKD1/2, 1.4 years in CKD3, and 0.5 years in CKD4 (Figure 3). Furthermore, sparsentan-treated patients spent an average of 10.6 years with ESRD, a reduction of 2.2 years in comparison with irbesartan (a reduction of 0.1 years, 1.3 years, and 0.8 years spent in pre-RRT, dialysis, and transplant health states, respectively). Improved survival and delayed disease progression translated to significant QALY gains for patients treated with sparsentan in comparison with irbesartan: a total gain of 16.9 QALYs or an incremental gain of 1.2 QALYs (11.4 QALYs total, 0.7 incremental QALYs gained discounted at 3.5% per annum).

## 4. Discussion

This study presented a novel lifetime health economic model that can be used to effectively quantify the health benefits for patients in treatment with sparsentan as a consequence of reduced UP/C and prolonged retention of kidney function. The model builds upon previous models of IgAN and CKD more generally, which have typically utilised either eGFR (based on CKD stage) or proteinuria in isolation. The new approach presented in this study allows for the model to capture both the definitive outcome of renal function while also reflecting the important prognostic value of UP/C on long-term health outcomes for people living with IgAN. Furthermore, as clinical trials for treatments of IgAN and other glomerulonephritis diseases are typically powered to identify improvements in proteinuria, this approach can be used to more effectively translate the findings of clinical trials, such as PROTECT, to health economic outcomes. The developed model was validated with real-world evidence and showed good concordance between model predictions and observed outcomes, with high R^2^ and low RMSE over a 15-year model horizon. The model was subsequently accepted for use in the NICE technology appraisal for sparsentan [13].

Based on the model analysis, sparsentan is anticipated to provide clinically meaningful improvements in outcomes for people with IgAN. The improvements in UP/C and eGFR observed in PROTECT translated to a delayed onset of ESRD, with 2.2 fewer years spent with late-stage disease in comparison with irbesartan. This resulted in increased total life years and QALYs gained in comparison with irbesartan of 0.9 years and 1.2 QALYs, respectively.

As with any modelling exercise, this study is associated with several limitations. First, the model presented is necessarily a simplification of the disease and patient experience and requires significant extrapolation beyond the follow-up of the PROTECT clinical trial. While model external validation showed that the predictions were generally consistent with observed real-world evidence, there were discrepancies. Notably, the model tended to overestimate ESRD-free survival in patients with UP/C 0.88 to <1.76 g/g from around year five onwards and tended to underestimate ESRD-free survival in patients with UP/C ≥ 1.76 g/g. The model also assumes that patients are homogeneous within health states. Although this assumption is necessary in the context of a Markov state transition model, the rate of eGFR decline for patients with IgAN can vary significantly, and in a non-linear disease such as IgAN, this assumption may cause the model to systematically under- or overestimate the incidence of ESRD. Finally, sodium glucose transport protein 2 (SGLT2) inhibitors are now recommended as part of standard of care treatment for IgAN and can be used in combination with sparsentan. PROTECT, and the long-term RaDaR dataset did not include data regarding treatment with concomitant SGLT2 inhibitors. And no direct comparative evidence for sparsentan and SGLT2 inhibitors is available. Consequently, the relative health benefits of sparsentan versus SGLT2 inhibitors are uncertain.

Future adaptations of this model could incorporate country-specific costs associated with disease management and HCRU and allow for a cost–utility analysis to assess if sparsentan is a cost-effective treatment for IgAN. This could build on the analysis presented by the National Institute for Health and Care Excellence in the UK, which concluded that sparsentan was a cost-effective treatment for IgAN based on the model presented in this study [13].

In conclusion, this study presents a new structure for health economic models in IgAN that more comprehensively captures the effect of proteinuria in combination with CKD progression. This new approach ultimately allows for the more robust implementation of clinical trial data in IgAN and, consequently, estimates of the cost-effectiveness of new treatments such as sparsentan. The analysis also quantified the impact of sparsentan, which has the potential to improve the impact of IgAN on patients by delaying disease progression and, consequently, improving patient health outcomes and HRQoL. Furthermore, by delaying the requirement for RRT, there is the potential to positively impact service delivery with significant anticipated cost-offsets to the acquisition cost of sparsentan.

## Figures and Tables

**Table 1:** Patient characteristics (N = 404).

Characteristic	Value
Age (mean)	46 years
Sex (male)	69.8%
Weight (mean)	84 kg
CKD1&2 (g/g 0- < 0.44)	1.24%
CKD3 (g/g 0- < 0.44)	1.24%
CKD4 (g/g 0- < 0.44)	0.25%
CKD1&2 (g/g 0.44- < 0.88)	9.16%
CKD3 (g/g 0.44- < 0.88)	15.10%
CKD4 (g/g 0.44- < 0.88)	1.24%
CKD1&2 (g/g 0.88- < 1.76)	18.32%
CKD3 (g/g 0.88- < 1.76)	25.25%
CKD4 (g/g 0.88- < 1.76)	1.98%
CKD1&2 (g/g >= 1.76)	7.92%
CKD3 (g/g >= 1.76)	16.83%
CKD4 (g/g >= 1.76)	1.49%

Abbreviations: CKD, chronic kidney disease. Percentages refer to the proportion of the overall population that fits into a specific category.

**Table 2:** KDIGO CKD stages used in the economic model.

CKD Stage	eGFR ^1^
CKD1, CKD2	≥60
CKD3	30–59
CKD4	15–29
CKD5 (ESRD)	<15

^1^ mL/min/1.73 m^2^; Abbreviations: CKD, chronic kidney disease; ESRD, end-stage renal disease.

**Table 3:** Disutilities applied to adverse events.

Adverse Event Type	Mean Disutility
Metabolism and nutrition disorders	0.00
Nervous system disorders	−0.07
Vascular disorders	−0.10
Investigations	0.00
General disorders and administration site conditions	0.00
Gastrointestinal disorders	−0.05
Renal and urinary disorders	−0.10

Adverse event types categorised according to System Organ Class.

**Table 4:** Mortality hazard ratios by CKD stage.

CKD Stage	Mortality HR
CKD1, CKD2	1.00
CKD3	1.55
CKD4	2.80
ESRD pre-RRT	4.60
ESRD dialysis ^1^	6.96
ESRD transplant	1.40

^1^ Values for dialysis comprised of weighted average of peritoneal dialysis (12.6%) and haemodialysis (87.4%), sourced from the UKRR. Abbreviations: CKD, chronic kidney disease; ESRD, end-stage renal disease; RRT, renal replacement therapy.

**Table 5:** Utility values reported in Cooper et al. [31].

CKD Stage	Mean	Standard Error
CKD1, CKD2	0.85	0.08
CKD3	0.80	0.08
CKD4	0.74	0.06
ESRD pre-RRT	0.44	0.01
ESRD dialysis ^1^	0.44	0.032
ESRD transplant	0.71	0.019

^1^ Values for dialysis comprised of weighted average of peritoneal dialysis (12.6%) and haemodialysis (87.4%), sourced from the UKKR. Abbreviations: CKD, chronic kidney disease; ESRD, end-stage renal disease; RRT, renal replacement therapy.

**Table 6:** Life year and quality-adjusted life year gains and declines.

	Sparsentan	Irbesartan	Incremental
	**Life years**		
Pre-ESRD	14.87	11.72	3.14
Pre-RRT	1.18	1.30	−0.12
Dialysis	6.15	7.49	−1.34
Transplant	3.26	4.04	−0.79
**Total**	**25.45**	**24.56**	**0.89**
		**Quality-adjusted life years**	
Pre-ESRD	8.43	6.95	1.48
Pre-RRT	0.82	0.92	−0.09
Dialysis	2.60	3.18	−0.58
Transplant	2.11	2.63	−0.52
AEs	−0.13	−0.08	−0.05
**Total**	**16.87**	**15.72**	**1.16**

Abbreviations: AE, adverse event; ESRD, end-stage renal disease; RRT, renal replacement therapy.

## Data Availability

The original contributions presented in this study are included in the article/Supplementary Materials. Further inquiries can be directed to the corresponding author.

## Supplementary Materials

The following supporting information can be downloaded at https://www.mdpi.com/article/10.3390/jcm15114201/s1: Document S1: Logistic regression weightings; Document S2: Transition matrices calculations; Figure S1: life year tornado plot; Figure S2: QALY tornado plot.

## Abbreviations

The following abbreviations are used in this manuscript:

AE	Adverse event
CKD	Chronic kidney disease
CONSORT	Consolidated Standards of Reporting Trials
ESRD	End-stage renal disease
HCRU	Healthcare resource utilisation
HR	Hazard ratio
HRQoL	Health-related quality of life
HTA	Health technology assessment
IgA	Immunoglobulin A
IgAN	IgA nephropathy
KDIGO	Kidney Disease: Improving Global Outcomes
NICE	National Institute of Health and Care Excellence
QALY	Quality-adjusted life year
QoL	Quality of life
RMSE	Root mean square error
RRT	Renal replacement therapy
TEAE	Treatment-emergent adverse event
UKRR	UK Renal Registry
UP/C	Urine protein/creatinine ratio
UPE	Urine protein excretion
