# Supplementary material for: Sparsentan for the Treatment of Immunoglobulin A Nephropathy: An Innovative Concept for Economic Modelling
Source: J Clin Med. 2026 May 29;15(11):4201. doi: 10.3390/jcm15114201 (PMC13258338; doi:10.3390/jcm15114201)
Supplement: Supplementary file 1 [file jcm-15-04201-s001.zip › File S1 logistic regression weightings.pdf]

Document S1 logistic regression weightings

Individual patients in the PROTECT aligned RaDaR IgAN cohort were assigned weights such that firstly, weighted mean baseline characteristics in the RaDaR cohort matched those reported for patients in the PROTECT trial, and secondly, each patient's weight was equal to their estimated odds of enrolment in the PROTECT trial versus the RaDaR cohort. Weights meeting these conditions were obtained based on a logistic regression model for the propensity of enrolment in the PROTECT trial versus the RaDaR IgAN cohort, with all matched-on baseline characteristics included as independent variables in the propensity score model. Denoting the weight for each RaDaR IgAN cohort patient as w_i, and the baseline characteristics vector for each RaDaR IgAN cohort patient as $\boldsymbol{X_i}$, the logistic regression model can be expressed as:

$$\log(w_i) = \alpha_0 + \alpha_1^T X_i$$

With summary statistics for baseline characteristics from the PROTECT patient population, a method of moments estimator was used to estimate the parameters of the logistic regression model. Distributions of weights were inspected to identify potential sensitivity to extreme weights, and the effective sample size computed as:

$$ESS = \frac{(\sum_{i=1}^{N} \widehat{w}_i)^2}{\sum_{i=1}^{N} \widehat{w}_i^2}$$

After matching, baseline characteristics of the derived RaDaR IgAN patient cohort were compared to PROTECT patient population characteristics after matching. P-values for continuous variables and categorical variables were calculated using Wald tests.

After applying weights obtained from the logistic regression model to the RaDaR IgAN cohort, differences in mean baseline characteristics between the weighted RaDaR IgAN cohort and the PROTECT trial population, approached zero (see table). The post-weighting effective sample size of the derived RaDaR IgAN patient cohort was 346 (representing 79% of the original, aligned to key PROTECT trial inclusion/exclusion criteria, population of 438 IgAN patients), indicative of good alignment in patient populations.

	**PROTECT aggregate**	**RaDaR unweighted**	**Difference unweighted**	**RaDaR weighted**	**Difference weighted**
Age, years, median (IQR)	46.0 (36.9, 55.9)	43.1 (32.5, 54.9)	-2.9	45.9 (36.9, 55.9)	-0.1
Male, n (%)	70%	67%	-3%	70%	0%
**Race, n (%)**					
White	67.0%	81.7%	14.7%	67%	0%
Asian	27.9%	14.6%	-13.3%	28%	.1%
Other	5.0%	3.7%	-1.3%	5%	0%
24-Hour PER, g/day, median (IQR)	1.79 (1.29, 2.76)	1.93 (1.34, 3.14)	0.14	1.80 (1.29, 2.76)	0.01
**CKD stage, n (%)**					
1/2	37%	39%	2%	37%	0%
3	58%	52%	-6%	58%	0%
4	5%	9%	4%	5%	0%

Abbreviations: CKD, chronic kidney disease; IQR, inter-quartile range; n, number; PER, protein excretion rate; RaDaR, National Registry of Rare Kidney Diseases
