# Supplementary material for: Sparsentan for the Treatment of Immunoglobulin A Nephropathy: An Innovative Concept for Economic Modelling
Source: J Clin Med. 2026 May 29;15(11):4201. doi: 10.3390/jcm15114201 (PMC13258338; doi:10.3390/jcm15114201)
Supplement: Supplementary file 1 [file jcm-15-04201-s001.zip › File S2-transition matrices calculations.pdf]

Documents S2 Transition matrix calculation – worked example

The TMs used in the model are constructed using data from several different sources: the PROTECT clinical trial, the RaDaR dataset, and the UK renal registry (UKRR). The following is a worked example of how these sources are combined to create the complete transition matrices identified in the above section.

Calculation starts with 4 individual 4x4 transition matrices outlining the transitions between CKD states, stratified by the patients UP/C banding (Table 1 to Table 4). These transitions are informed by the PROTECT trial and RaDaR dataset and are treatment agnostic. Transitions differ depending on the patients UP/C banding, for example the probability that a patient moves from CKD3 to CKD4 with a UP/C level <0.44 g/g denoted as $\boldsymbol{P_1(3 \rightarrow 4)}$ does not equal the probability that a patient moves from CKD3 to CKD4 with a UP/C level 0.44-<0.88 g/g denoted as $\boldsymbol{P_2(3 \rightarrow 4)}$. Ie:

$$\boldsymbol{P_1(3 \rightarrow 4) \neq P_2(3 \rightarrow 4) \neq P_3(3 \rightarrow 4) \neq P_4(3 \rightarrow 4)}$$

Table 1: CKD Transitions 1 - UP/C <0.44 g/g

	CKD1&2	CKD3	CKD4	CKD5
**CKD1&2**	$P_1(1\&2 \rightarrow 1\&2)$	$P_1(1\&2 \rightarrow 3)$	$P_1(1\&2 \rightarrow 4)$	$P_1(1\&2 \rightarrow 5)$
**CKD3**	$P_1(3 \rightarrow 1\&2)$	$P_1(3 \rightarrow 3)$	$P_1(3 \rightarrow 4)$	$P_1(3 \rightarrow 5)$
**CKD4**	$P_1(4 \rightarrow 1\&2)$	$P_1(4 \rightarrow 3)$	$P_1(4 \rightarrow 4)$	$P_1(4 \rightarrow 5)$
**CKD5**	$P_1(5 \rightarrow 1\&2)$	$P_1(5 \rightarrow 3)$	$P_1(5 \rightarrow 4)$	$P_1(5 \rightarrow 5)$

Table 2: CKD Transitions 2 - UP/C 0.44-<0.88 g/g

	CKD1&2	CKD3	CKD4	CKD5
**CKD1&2**	$P_2(1\&2 \rightarrow 1\&2)$	$P_2(1\&2 \rightarrow 3)$	$P_2(1\&2 \rightarrow 4)$	$P_2(1\&2 \rightarrow 5)$
**CKD3**	$P_2(3 \rightarrow 1\&2)$	$P_2(3 \rightarrow 3)$	$P_2(3 \rightarrow 4)$	$P_2(3 \rightarrow 5)$
**CKD4**	$P_2(4 \rightarrow 1\&2)$	$P_2(4 \rightarrow 3)$	$P_2(4 \rightarrow 4)$	$P_2(4 \rightarrow 5)$
**CKD5**	$P_2(5 \rightarrow 1\&2)$	$P_2(5 \rightarrow 3)$	$P_2(5 \rightarrow 4)$	$P_2(5 \rightarrow 5)$

Table 3: CKD Transitions 3 - UP/C 0.88-<1.76 g/g

	CKD1&2	CKD3	CKD4	CKD5
**CKD1&2**	$P_3(1\&2 \rightarrow 1\&2)$	$P_3(1\&2 \rightarrow 3)$	$P_3(1\&2 \rightarrow 4)$	$P_3(1\&2 \rightarrow 5)$
**CKD3**	$P_3(3 \rightarrow 1\&2)$	$P_3(3 \rightarrow 3)$	$P_3(3 \rightarrow 4)$	$P_3(3 \rightarrow 5)$
**CKD4**	$P_3(4 \rightarrow 1\&2)$	$P_3(4 \rightarrow 3)$	$P_3(4 \rightarrow 4)$	$P_3(4 \rightarrow 5)$
**CKD5**	$P_3(5 \rightarrow 1\&2)$	$P_3(5 \rightarrow 3)$	$P_3(5 \rightarrow 4)$	$P_3(5 \rightarrow 5)$

Table 4: CKD Transitions 4 - UP/C >=1.76 g/g

	CKD1&2	CKD3	CKD4	CKD5
**CKD1&2**	$P_4(1\&2 \rightarrow 1\&2)$	$P_4(1\&2 \rightarrow 3)$	$P_4(1\&2 \rightarrow 4)$	$P_4(1\&2 \rightarrow 5)$
**CKD3**	$P_4(3 \rightarrow 1\&2)$	$P_4(3 \rightarrow 3)$	$P_4(3 \rightarrow 4)$	$P_4(3 \rightarrow 5)$
**CKD4**	$P_4(4 \rightarrow 1\&2)$	$P_4(4 \rightarrow 3)$	$P_4(4 \rightarrow 4)$	$P_4(4 \rightarrow 5)$
**CKD5**	$P_4(5 \rightarrow 1\&2)$	$P_4(5 \rightarrow 3)$	$P_4(5 \rightarrow 4)$	$P_4(5 \rightarrow 5)$

In a similar structure, patients have a single 4x4 transition matrix for UP/C transitions (Table 5), informed by observations of movements in the PROTECT trial. These transitions are treatment specific, and as such there are different transitions for each treatment arm. For the purposes of this worked example, the calculations focus on calculations for one treatment arm only.

Table 5: UP/C Transitions

	<0.44 g/g	0.44-<0.88 g/g	0.88-<1.76 g/g	>=1.76 g/g
**<0.44 g/g**	Q(1→1)	Q(1→2)	Q(1→3)	Q(1→4)
**0.44-<0.88 g/g**	Q(2→1)	Q(2→2)	Q(2→3)	Q(2→4)
**0.88-<1.76 g/g**	Q(3→1)	Q(3→2)	Q(3→3)	Q(3→4)
**>=1.76 g/g**	Q(4→1)	Q(4→2)	Q(4→3)	Q(4→4)

This TM for UP/C is applied multiplicatively to the four TMs for CKD to create the following outlined in Table 6:

Table 6: CKD transitions by UP/C (CKD1-4 only)

		g/g <0.44			g/g 0.44-<0.88			g/g 0.88-<1.76			g/g >=1.76		
		CKD1&2	CKD3	CKD4	CKD1&2	CKD3	CKD4	CKD1&2	CKD3	CKD4	CKD1&2	CKD3	CKD4
g/g <0.44	CKD1&2	P_1(1&2→1&2) × Q(1→1)	P_1(1&2→3) × Q(1→1)	P_1(1&2→4) × Q(1→1)	P_1(1&2→1&2) × Q(1→2)	P_1(1&2→3) × Q(1→2)	P_1(1&2→4) × Q(1→2)	P_1(1&2→1&2) × Q(1→3)	P_1(1&2→3) × Q(1→3)	P_1(1&2→4) × Q(1→3)	P_1(1&2→1&2) × Q(1→4)	P_1(1&2→3) × Q(1→4)	P_1(1&2→4) × Q(1→4)
	CKD3	P_1(3→1&2) × Q(1→1)	P_1(3→3) × Q(1→1)	P_1(3→4) × Q(1→1)	P_1(3→1&2) × Q(1→2)	P_1(3→3) × Q(1→2)	P_1(3→4) × Q(1→2)	P_1(3→1&2) × Q(1→3)	P_1(3→3) × Q(1→3)	P_1(3→4) × Q(1→3)	P_1(3→1&2) × Q(1→4)	P_1(3→3) × Q(1→4)	P_1(3→4) × Q(1→4)
	CKD4	P_1(4→1&2) × Q(1→1)	P_1(4→3) × Q(1→1)	P_1(4→4) × Q(1→1)	P_1(4→1&2) × Q(1→2)	P_1(4→3) × Q(1→2)	P_1(4→4) × Q(1→2)	P_1(4→1&2) × Q(1→3)	P_1(4→3) × Q(1→3)	P_1(4→4) × Q(1→3)	P_1(4→1&2) × Q(1→4)	P_1(4→3) × Q(1→4)	P_1(4→4) × Q(1→4)
g/g 0.44-<0.88	CKD1&2	P_2(1&2→1&2) × Q(2→1)	P_2(1&2→3) × Q(2→1)	P_2(1&2→4) × Q(2→1)	P_2(1&2→1&2) × Q(2→2)	P_2(1&2→3) × Q(2→2)	P_2(1&2→4) × Q(2→2)	P_2(1&2→1&2) × Q(2→3)	P_2(1&2→3) × Q(2→3)	P_2(1&2→4) × Q(2→3)	P_2(1&2→1&2) × Q(2→4)	P_2(1&2→3) × Q(2→4)	P_2(1&2→4) × Q(2→4)
	CKD3	P_2(3→1&2) × Q(2→1)	P_2(3→3) × Q(2→1)	P_2(3→4) × Q(2→1)	P_2(3→1&2) × Q(2→2)	P_2(3→3) × Q(2→2)	P_2(3→4) × Q(2→2)	P_2(3→1&2) × Q(2→3)	P_2(3→3) × Q(2→3)	P_2(3→4) × Q(2→3)	P_2(3→1&2) × Q(2→4)	P_2(3→3) × Q(2→4)	P_2(3→4) × Q(2→4)
	CKD4	P_2(4→1&2) × Q(2→1)	P_2(4→3) × Q(2→1)	P_2(4→4) × Q(2→1)	P_2(4→1&2) × Q(2→2)	P_2(4→3) × Q(2→2)	P_2(4→4) × Q(2→2)	P_2(4→1&2) × Q(2→3)	P_2(4→3) × Q(2→3)	P_2(4→4) × Q(2→3)	P_2(4→1&2) × Q(2→4)	P_2(4→3) × Q(2→4)	P_2(4→4) × Q(2→4)
g/g 0.88-<1.76	CKD1&2	P_3(1&2→1&2) × Q(3→1)	P_3(1&2→3) × Q(3→1)	P_3(1&2→4) × Q(3→1)	P_3(1&2→1&2) × Q(3→2)	P_3(1&2→3) × Q(3→2)	P_3(1&2→4) × Q(3→2)	P_3(1&2→1&2) × Q(3→3)	P_3(1&2→3) × Q(3→3)	P_3(1&2→4) × Q(3→3)	P_3(1&2→1&2) × Q(3→4)	P_3(1&2→3) × Q(3→4)	P_3(1&2→4) × Q(3→4)
	CKD3	P_3(3→1&2) × Q(3→1)	P_3(3→3) × Q(3→1)	P_3(3→4) × Q(3→1)	P_3(3→1&2) × Q(3→2)	P_3(3→3) × Q(3→2)	P_3(3→4) × Q(3→2)	P_3(3→1&2) × Q(3→3)	P_3(3→3) × Q(3→3)	P_3(3→4) × Q(3→3)	P_3(3→1&2) × Q(3→4)	P_3(3→3) × Q(3→4)	P_3(3→4) × Q(3→4)
	CKD4	P_3(4→1&2) × Q(3→1)	P_3(4→3) × Q(3→1)	P_3(4→4) × Q(3→1)	P_3(4→1&2) × Q(3→2)	P_3(4→3) × Q(3→2)	P_3(4→4) × Q(3→2)	P_3(4→1&2) × Q(3→3)	P_3(4→3) × Q(3→3)	P_3(4→4) × Q(3→3)	P_3(4→1&2) × Q(3→4)	P_3(4→3) × Q(3→4)	P_3(4→4) × Q(3→4)
g/g >= 1.76	CKD1&2	P_4(1&2→1&2) × Q(4→1)	P_4(1&2→3) × Q(4→1)	P_4(1&2→4) × Q(4→1)	P_4(1&2→1&2) × Q(4→2)	P_4(1&2→3) × Q(4→2)	P_4(1&2→4) × Q(4→2)	P_4(1&2→1&2) × Q(4→3)	P_4(1&2→3) × Q(4→3)	P_4(1&2→4) × Q(4→3)	P_4(1&2→1&2) × Q(4→4)	P_4(1&2→3) × Q(4→4)	P_4(1&2→4) × Q(4→4)
	CKD3	P_4(3→1&2) × Q(4→1)	P_4(3→3) × Q(4→1)	P_4(3→4) × Q(4→1)	P_4(3→1&2) × Q(4→2)	P_4(3→3) × Q(4→2)	P_4(3→4) × Q(4→2)	P_4(3→1&2) × Q(4→3)	P_4(3→3) × Q(4→3)	P_4(3→4) × Q(4→3)	P_4(3→1&2) × Q(4→4)	P_4(3→3) × Q(4→4)	P_4(3→4) × Q(4→4)
	CKD4	P_4(4→1&2) × Q(4→1)	P_4(4→3) × Q(4→1)	P_4(4→4) × Q(4→1)	P_4(4→1&2) × Q(4→2)	P_4(4→3) × Q(4→2)	P_4(4→4) × Q(4→2)	P_4(4→1&2) × Q(4→3)	P_4(4→3) × Q(4→3)	P_4(4→4) × Q(4→3)	P_4(4→1&2) × Q(4→4)	P_4(4→3) × Q(4→4)	P_4(4→4) × Q(4→4)

Note that above in Table 6, transitions to CKD5, i.e. end-stage renal disease (ESRD), have not been included. This is because at CKD5 patients are aggregated together regardless of UP/C band as this is no longer a driving factor of the disease. Patients are assumed to remain in the CKD5 state once they enter, leading to the following TM outlined in Table 7.

Table 7: CKD transitions by UP/C (CKD1-5)

		g/g <0.44			g/g 0.44-<0.88			g/g 0.88-<1.76			g/g >=1.76			ESRD (CKD5)
		CKD1&2	CKD3	CKD4	CKD1&2	CKD3	CKD4	CKD1&2	CKD3	CKD4	CKD1&2	CKD3	CKD4	
g/g <0.44	CKD 1&2	P_1(1&2→1&2) × Q(1→1)	P_1(1&2→3) × Q(1→1)	P_1(1&2→4) × Q(1→1)	P_1(1&2→1&2) × Q(1→2)	P_1(1&2→3) × Q(1→2)	P_1(1&2→4) × Q(1→2)	P_1(1&2→1&2) × Q(1→3)	P_1(1&2→3) × Q(1→3)	P_1(1&2→4) × Q(1→3)	P_1(1&2→1&2) × Q(1→4)	P_1(1&2→3) × Q(1→4)	P_1(1&2→4) × Q(1→4)	P_1(1&2→5)
	CKD3	P_1(3→1&2) × Q(1→1)	P_1(3→3) × Q(1→1)	P_1(3→4) × Q(1→1)	P_1(3→1&2) × Q(1→2)	P_1(3→3) × Q(1→2)	P_1(3→4) × Q(1→2)	P_1(3→1&2) × Q(1→3)	P_1(3→3) × Q(1→3)	P_1(3→4) × Q(1→3)	P_1(3→1&2) × Q(1→4)	P_1(3→3) × Q(1→4)	P_1(3→4) × Q(1→4)	P_1(3→5)
	CKD4	P_1(4→1&2) × Q(1→1)	P_1(4→3) × Q(1→1)	P_1(4→4) × Q(1→1)	P_1(4→1&2) × Q(1→2)	P_1(4→3) × Q(1→2)	P_1(4→4) × Q(1→2)	P_1(4→1&2) × Q(1→3)	P_1(4→3) × Q(1→3)	P_1(4→4) × Q(1→3)	P_1(4→1&2) × Q(1→4)	P_1(4→3) × Q(1→4)	P_1(4→4) × Q(1→4)	P_1(4→5)
g/g 0.44-<0.88	CKD 1&2	P_2(1&2→1&2) × Q(2→1)	P_2(1&2→3) × Q(2→1)	P_2(1&2→4) × Q(2→1)	P_2(1&2→1&2) × Q(2→2)	P_2(1&2→3) × Q(2→2)	P_2(1&2→4) × Q(2→2)	P_2(1&2→1&2) × Q(2→3)	P_2(1&2→3) × Q(2→3)	P_2(1&2→4) × Q(2→3)	P_2(1&2→1&2) × Q(2→4)	P_2(1&2→3) × Q(2→4)	P_2(1&2→4) × Q(2→4)	P_2(1&2→5)
	CKD3	P_2(3→1&2) × Q(2→1)	P_2(3→3) × Q(2→1)	P_2(3→4) × Q(2→1)	P_2(3→1&2) × Q(2→2)	P_2(3→3) × Q(2→2)	P_2(3→4) × Q(2→2)	P_2(3→1&2) × Q(2→3)	P_2(3→3) × Q(2→3)	P_2(3→4) × Q(2→3)	P_2(3→1&2) × Q(2→4)	P_2(3→3) × Q(2→4)	P_2(3→4) × Q(2→4)	P_2(3→5)
	CKD4	P_2(4→1&2) × Q(2→1)	P_2(4→3) × Q(2→1)	P_2(4→4) × Q(2→1)	P_2(4→1&2) × Q(2→2)	P_2(4→3) × Q(2→2)	P_2(4→4) × Q(2→2)	P_2(4→1&2) × Q(2→3)	P_2(4→3) × Q(2→3)	P_2(4→4) × Q(2→3)	P_2(4→1&2) × Q(2→4)	P_2(4→3) × Q(2→4)	P_2(4→4) × Q(2→4)	P_2(4→5)
g/g 0.88-<1.76	CKD 1&2	P_3(1&2→1&2) × Q(3→1)	P_3(1&2→3) × Q(3→1)	P_3(1&2→4) × Q(3→1)	P_3(1&2→1&2) × Q(3→2)	P_3(1&2→3) × Q(3→2)	P_3(1&2→4) × Q(3→2)	P_3(1&2→1&2) × Q(3→3)	P_3(1&2→3) × Q(3→3)	P_3(1&2→4) × Q(3→3)	P_3(1&2→1&2) × Q(3→4)	P_3(1&2→3) × Q(3→4)	P_3(1&2→4) × Q(3→4)	P_3(1&2→5)
	CKD3	P_3(3→1&2) × Q(3→1)	P_3(3→3) × Q(3→1)	P_3(3→4) × Q(3→1)	P_3(3→1&2) × Q(3→2)	P_3(3→3) × Q(3→2)	P_3(3→4) × Q(3→2)	P_3(3→1&2) × Q(3→3)	P_3(3→3) × Q(3→3)	P_3(3→4) × Q(3→3)	P_3(3→1&2) × Q(3→4)	P_3(3→3) × Q(3→4)	P_3(3→4) × Q(3→4)	P_3(3→5)
	CKD4	P_3(4→1&2) × Q(3→1)	P_3(4→3) × Q(3→1)	P_3(4→4) × Q(3→1)	P_3(4→1&2) × Q(3→2)	P_3(4→3) × Q(3→2)	P_3(4→4) × Q(3→2)	P_3(4→1&2) × Q(3→3)	P_3(4→3) × Q(3→3)	P_3(4→4) × Q(3→3)	P_3(4→1&2) × Q(3→4)	P_3(4→3) × Q(3→4)	P_3(4→4) × Q(3→4)	P_3(4→5)
g/g >= 1.76	CKD 1&2	P_4(1&2→1&2) × Q(4→1)	P_4(1&2→3) × Q(4→1)	P_4(1&2→4) × Q(4→1)	P_4(1&2→1&2) × Q(4→2)	P_4(1&2→3) × Q(4→2)	P_4(1&2→4) × Q(4→2)	P_4(1&2→1&2) × Q(4→3)	P_4(1&2→3) × Q(4→3)	P_4(1&2→4) × Q(4→3)	P_4(1&2→1&2) × Q(4→4)	P_4(1&2→3) × Q(4→4)	P_4(1&2→4) × Q(4→4)	P_3(1&2→5)
	CKD3	P_4(3→1&2) × Q(4→1)	P_4(3→3) × Q(4→1)	P_4(3→4) × Q(4→1)	P_4(3→1&2) × Q(4→2)	P_4(3→3) × Q(4→2)	P_4(3→4) × Q(4→2)	P_4(3→1&2) × Q(4→3)	P_4(3→3) × Q(4→3)	P_4(3→4) × Q(4→3)	P_4(3→1&2) × Q(4→4)	P_4(3→3) × Q(4→4)	P_4(3→4) × Q(4→4)	P_3(3→5)
	CKD4	P_4(4→1&2) × Q(4→1)	P_4(4→3) × Q(4→1)	P_4(4→4) × Q(4→1)	P_4(4→1&2) × Q(4→2)	P_4(4→3) × Q(4→2)	P_4(4→4) × Q(4→2)	P_4(4→1&2) × Q(4→3)	P_4(4→3) × Q(4→3)	P_4(4→4) × Q(4→3)	P_4(4→1&2) × Q(4→4)	P_4(4→3) × Q(4→4)	P_4(4→4) × Q(4→4)	P_3(4→5)
ESRD (CKD5)		0%	0%	0%	0%	0%	0%	0%	0%	0%	0%	0%	0%	100%

Within ESRD, 3 states are defined: pre-RRT (renal-replacement therapy), dialysis, and transplant (Table 8). Transitions between these states are informed by the UKRR. It is assumed that the initial distribution of patients moving into the ESRD state for the first time matches the probability of moving from the pre-RRT state to the respective state (Table 9).

Table 8: ESRD transitions

	**Pre-RRT**	**Dialysis**	**Transplant**
**Pre-RRT**	R(1→1)	R(1→2)	R(1→3)
**Dialysis**	R(2→1)	R(2→2)	R(2→3)
**Transplant**	R(3→1)	R(3→2)	R(3→3)

Table 9: Initial ESRD transitions (from earlier CKD states)

	**Pre-RRT**	**Dialysis**	**Transplant**
**CKD1-4**	R(1→1)	R(1→2)	R(1→3)

Hence, Table 10 below illustrates the complete structure of the transition matrices used in the model.

Table 10: CKD transitions by UP/C (CKD1-5)

		g/g <0.44			g/g 0.44-<0.88			g/g 0.88-<1.76			g/g >=1.76			ESRD (CKD5)		
		CKD1&2	CKD3	CKD4	CKD1&2	CKD3	CKD4	CKD1&2	CKD3	CKD4	CKD1&2	CKD3	CKD4	Pre-RRT	Dialysis	Transplant
g/g <0.44	CKD 1&2	$P_1(1\&2\rightarrow1\&2) \times Q(1\rightarrow1)$	$P_1(1\&2\rightarrow3) \times Q(1\rightarrow1)$	$P_1(1\&2\rightarrow4) \times Q(1\rightarrow1)$	$P_1(1\&2\rightarrow1\&2) \times Q(1\rightarrow2)$	$P_1(1\&2\rightarrow3) \times Q(1\rightarrow2)$	$P_1(1\&2\rightarrow4) \times Q(1\rightarrow2)$	$P_1(1\&2\rightarrow1\&2) \times Q(1\rightarrow3)$	$P_1(1\&2\rightarrow3) \times Q(1\rightarrow3)$	$P_1(1\&2\rightarrow4) \times Q(1\rightarrow3)$	$P_1(1\&2\rightarrow1\&2) \times Q(1\rightarrow4)$	$P_1(1\&2\rightarrow3) \times Q(1\rightarrow4)$	$P_1(1\&2\rightarrow4) \times Q(1\rightarrow4)$	$P_1(1\&2\rightarrow5) \times R(1\rightarrow1)$	$P_1(1\&2\rightarrow5) \times R(1\rightarrow2)$	$P_1(1\&2\rightarrow5) \times R(1\rightarrow3)$
	CKD3	$P_1(3\rightarrow1\&2) \times Q(1\rightarrow1)$	$P_1(3\rightarrow3) \times Q(1\rightarrow1)$	$P_1(3\rightarrow4) \times Q(1\rightarrow1)$	$P_1(3\rightarrow1\&2) \times Q(1\rightarrow2)$	$P_1(3\rightarrow3) \times Q(1\rightarrow2)$	$P_1(3\rightarrow4) \times Q(1\rightarrow2)$	$P_1(3\rightarrow1\&2) \times Q(1\rightarrow3)$	$P_1(3\rightarrow3) \times Q(1\rightarrow3)$	$P_1(3\rightarrow4) \times Q(1\rightarrow3)$	$P_1(3\rightarrow1\&2) \times Q(1\rightarrow4)$	$P_1(3\rightarrow3) \times Q(1\rightarrow4)$	$P_1(3\rightarrow4) \times Q(1\rightarrow4)$	$P_1(3\rightarrow5) \times R(1\rightarrow1)$	$P_1(3\rightarrow5) \times R(1\rightarrow2)$	$P_1(3\rightarrow5) \times R(1\rightarrow3)$
	CKD4	$P_1(4\rightarrow1\&2) \times Q(1\rightarrow1)$	$P_1(4\rightarrow3) \times Q(1\rightarrow1)$	$P_1(4\rightarrow4) \times Q(1\rightarrow1)$	$P_1(4\rightarrow1\&2) \times Q(1\rightarrow2)$	$P_1(4\rightarrow3) \times Q(1\rightarrow2)$	$P_1(4\rightarrow4) \times Q(1\rightarrow2)$	$P_1(4\rightarrow1\&2) \times Q(1\rightarrow3)$	$P_1(4\rightarrow3) \times Q(1\rightarrow3)$	$P_1(4\rightarrow4) \times Q(1\rightarrow3)$	$P_1(4\rightarrow1\&2) \times Q(1\rightarrow4)$	$P_1(4\rightarrow3) \times Q(1\rightarrow4)$	$P_1(4\rightarrow4) \times Q(1\rightarrow4)$	$P_1(4\rightarrow5) \times R(1\rightarrow1)$	$P_1(4\rightarrow5) \times R(1\rightarrow2)$	$P_1(4\rightarrow5) \times R(1\rightarrow3)$
g/g 0.44-<0.88	CKD 1&2	$P_2(1\&2\rightarrow1\&2) \times Q(2\rightarrow1)$	$P_2(1\&2\rightarrow3) \times Q(2\rightarrow1)$	$P_2(1\&2\rightarrow4) \times Q(2\rightarrow1)$	$P_2(1\&2\rightarrow1\&2) \times Q(2\rightarrow2)$	$P_2(1\&2\rightarrow3) \times Q(2\rightarrow2)$	$P_2(1\&2\rightarrow4) \times Q(2\rightarrow2)$	$P_2(1\&2\rightarrow1\&2) \times Q(2\rightarrow3)$	$P_2(1\&2\rightarrow3) \times Q(2\rightarrow3)$	$P_2(1\&2\rightarrow4) \times Q(2\rightarrow3)$	$P_2(1\&2\rightarrow1\&2) \times Q(2\rightarrow4)$	$P_2(1\&2\rightarrow3) \times Q(2\rightarrow4)$	$P_2(1\&2\rightarrow4) \times Q(2\rightarrow4)$	$P_2(1\&2\rightarrow5) \times R(1\rightarrow1)$	$P_2(1\&2\rightarrow5) \times R(1\rightarrow2)$	$P_2(1\&2\rightarrow5) \times R(1\rightarrow3)$
	CKD3	$P_2(3\rightarrow1\&2) \times Q(2\rightarrow1)$	$P_2(3\rightarrow3) \times Q(2\rightarrow1)$	$P_2(3\rightarrow4) \times Q(2\rightarrow1)$	$P_2(3\rightarrow1\&2) \times Q(2\rightarrow2)$	$P_2(3\rightarrow3) \times Q(2\rightarrow2)$	$P_2(3\rightarrow4) \times Q(2\rightarrow2)$	$P_2(3\rightarrow1\&2) \times Q(2\rightarrow3)$	$P_2(3\rightarrow3) \times Q(2\rightarrow3)$	$P_2(3\rightarrow4) \times Q(2\rightarrow3)$	$P_2(3\rightarrow1\&2) \times Q(2\rightarrow4)$	$P_2(3\rightarrow3) \times Q(2\rightarrow4)$	$P_2(3\rightarrow4) \times Q(2\rightarrow4)$	$P_2(3\rightarrow5) \times R(1\rightarrow1)$	$P_2(3\rightarrow5) \times R(1\rightarrow2)$	$P_2(3\rightarrow5) \times R(1\rightarrow3)$
	CKD4	$P_2(4\rightarrow1\&2) \times Q(2\rightarrow1)$	$P_2(4\rightarrow3) \times Q(2\rightarrow1)$	$P_2(4\rightarrow4) \times Q(2\rightarrow1)$	$P_2(4\rightarrow1\&2) \times Q(2\rightarrow2)$	$P_2(4\rightarrow3) \times Q(2\rightarrow2)$	$P_2(4\rightarrow4) \times Q(2\rightarrow2)$	$P_2(4\rightarrow1\&2) \times Q(2\rightarrow3)$	$P_2(4\rightarrow3) \times Q(2\rightarrow3)$	$P_2(4\rightarrow4) \times Q(2\rightarrow3)$	$P_2(4\rightarrow1\&2) \times Q(2\rightarrow4)$	$P_2(4\rightarrow3) \times Q(2\rightarrow4)$	$P_2(4\rightarrow4) \times Q(2\rightarrow4)$	$P_2(4\rightarrow5) \times R(1\rightarrow1)$	$P_2(4\rightarrow5) \times R(1\rightarrow2)$	$P_2(4\rightarrow5) \times R(1\rightarrow3)$
g/g 0.88-<1.76	CKD 1&2	$P_3(1\&2\rightarrow1\&2) \times Q(3\rightarrow1)$	$P_3(1\&2\rightarrow3) \times Q(3\rightarrow1)$	$P_3(1\&2\rightarrow4) \times Q(3\rightarrow1)$	$P_3(1\&2\rightarrow1\&2) \times Q(3\rightarrow2)$	$P_3(1\&2\rightarrow3) \times Q(3\rightarrow2)$	$P_3(1\&2\rightarrow4) \times Q(3\rightarrow2)$	$P_3(1\&2\rightarrow1\&2) \times Q(3\rightarrow3)$	$P_3(1\&2\rightarrow3) \times Q(3\rightarrow3)$	$P_3(1\&2\rightarrow4) \times Q(3\rightarrow3)$	$P_3(1\&2\rightarrow1\&2) \times Q(3\rightarrow4)$	$P_3(1\&2\rightarrow3) \times Q(3\rightarrow4)$	$P_3(1\&2\rightarrow4) \times Q(3\rightarrow4)$	$P_3(1\&2\rightarrow5) \times R(1\rightarrow1)$	$P_3(1\&2\rightarrow5) \times R(1\rightarrow2)$	$P_3(1\&2\rightarrow5) \times R(1\rightarrow3)$
	CKD3	$P_3(3\rightarrow1\&2) \times Q(3\rightarrow1)$	$P_3(3\rightarrow3) \times Q(3\rightarrow1)$	$P_3(3\rightarrow4) \times Q(3\rightarrow1)$	$P_3(3\rightarrow1\&2) \times Q(3\rightarrow2)$	$P_3(3\rightarrow3) \times Q(3\rightarrow2)$	$P_3(3\rightarrow4) \times Q(3\rightarrow2)$	$P_3(3\rightarrow1\&2) \times Q(3\rightarrow3)$	$P_3(3\rightarrow3) \times Q(3\rightarrow3)$	$P_3(3\rightarrow4) \times Q(3\rightarrow3)$	$P_3(3\rightarrow1\&2) \times Q(3\rightarrow4)$	$P_3(3\rightarrow3) \times Q(3\rightarrow4)$	$P_3(3\rightarrow4) \times Q(3\rightarrow4)$	$P_3(3\rightarrow5) \times R(1\rightarrow1)$	$P_3(3\rightarrow5) \times R(1\rightarrow2)$	$P_3(3\rightarrow5) \times R(1\rightarrow3)$
	CKD4	$P_3(4\rightarrow1\&2) \times Q(3\rightarrow1)$	$P_3(4\rightarrow3) \times Q(3\rightarrow1)$	$P_3(4\rightarrow4) \times Q(3\rightarrow1)$	$P_3(4\rightarrow1\&2) \times Q(3\rightarrow2)$	$P_3(4\rightarrow3) \times Q(3\rightarrow2)$	$P_3(4\rightarrow4) \times Q(3\rightarrow2)$	$P_3(4\rightarrow1\&2) \times Q(3\rightarrow3)$	$P_3(4\rightarrow3) \times Q(3\rightarrow3)$	$P_3(4\rightarrow4) \times Q(3\rightarrow3)$	$P_3(4\rightarrow1\&2) \times Q(3\rightarrow4)$	$P_3(4\rightarrow3) \times Q(3\rightarrow4)$	$P_3(4\rightarrow4) \times Q(3\rightarrow4)$	$P_3(4\rightarrow5) \times R(1\rightarrow1)$	$P_3(4\rightarrow5) \times R(1\rightarrow2)$	$P_3(4\rightarrow5) \times R(1\rightarrow3)$
g/g >= 1.76	CKD 1&2	$P_4(1\&2\rightarrow1\&2) \times Q(4\rightarrow1)$	$P_4(1\&2\rightarrow3) \times Q(4\rightarrow1)$	$P_4(1\&2\rightarrow4) \times Q(4\rightarrow1)$	$P_4(1\&2\rightarrow1\&2) \times Q(4\rightarrow2)$	$P_4(1\&2\rightarrow3) \times Q(4\rightarrow2)$	$P_4(1\&2\rightarrow4) \times Q(4\rightarrow2)$	$P_4(1\&2\rightarrow1\&2) \times Q(4\rightarrow3)$	$P_4(1\&2\rightarrow3) \times Q(4\rightarrow3)$	$P_4(1\&2\rightarrow4) \times Q(4\rightarrow3)$	$P_4(1\&2\rightarrow1\&2) \times Q(4\rightarrow4)$	$P_4(1\&2\rightarrow3) \times Q(4\rightarrow4)$	$P_4(1\&2\rightarrow4) \times Q(4\rightarrow4)$	$P_3(1\&2\rightarrow5) \times R(1\rightarrow1)$	$P_3(1\&2\rightarrow5) \times R(1\rightarrow2)$	$P_3(1\&2\rightarrow5) \times R(1\rightarrow3)$
	CKD3	$P_4(3\rightarrow1\&2) \times Q(4\rightarrow1)$	$P_4(3\rightarrow3) \times Q(4\rightarrow1)$	$P_4(3\rightarrow4) \times Q(4\rightarrow1)$	$P_4(3\rightarrow1\&2) \times Q(4\rightarrow2)$	$P_4(3\rightarrow3) \times Q(4\rightarrow2)$	$P_4(3\rightarrow4) \times Q(4\rightarrow2)$	$P_4(3\rightarrow1\&2) \times Q(4\rightarrow3)$	$P_4(3\rightarrow3) \times Q(4\rightarrow3)$	$P_4(3\rightarrow4) \times Q(4\rightarrow3)$	$P_4(3\rightarrow1\&2) \times Q(4\rightarrow4)$	$P_4(3\rightarrow3) \times Q(4\rightarrow4)$	$P_4(3\rightarrow4) \times Q(4\rightarrow4)$	$P_3(3\rightarrow5) \times R(1\rightarrow1)$	$P_3(3\rightarrow5) \times R(1\rightarrow2)$	$P_3(3\rightarrow5) \times R(1\rightarrow3)$
	CKD4	$P_4(4\rightarrow1\&2) \times Q(4\rightarrow1)$	$P_4(4\rightarrow3) \times Q(4\rightarrow1)$	$P_4(4\rightarrow4) \times Q(4\rightarrow1)$	$P_4(4\rightarrow1\&2) \times Q(4\rightarrow2)$	$P_4(4\rightarrow3) \times Q(4\rightarrow2)$	$P_4(4\rightarrow4) \times Q(4\rightarrow2)$	$P_4(4\rightarrow1\&2) \times Q(4\rightarrow3)$	$P_4(4\rightarrow3) \times Q(4\rightarrow3)$	$P_4(4\rightarrow4) \times Q(4\rightarrow3)$	$P_4(4\rightarrow1\&2) \times Q(4\rightarrow4)$	$P_4(4\rightarrow3) \times Q(4\rightarrow4)$	$P_4(4\rightarrow4) \times Q(4\rightarrow4)$	$P_3(4\rightarrow5) \times R(1\rightarrow1)$	$P_3(4\rightarrow5) \times R(1\rightarrow2)$	$P_3(4\rightarrow5) \times R(1\rightarrow3)$
ESRD (CKD5)	Pre-RRT	0%	0%	0%	0%	0%	0%	0%	0%	0%	0%	0%	0%	$R(1\rightarrow1)$	$R(1\rightarrow2)$	$R(1\rightarrow3)$
	Dialysis	0%	0%	0%	0%	0%	0%	0%	0%	0%	0%	0%	0%	$R(2\rightarrow1)$	$R(2\rightarrow2)$	$R(2\rightarrow3)$
	Transplant	0%	0%	0%	0%	0%	0%	0%	0%	0%	0%	0%	0%	$R(3\rightarrow1)$	$R(3\rightarrow2)$	$R(3\rightarrow3)$
