# Supplementary figures and images for: Sparsentan for the Treatment of Immunoglobulin A Nephropathy: An Innovative Concept for Economic Modelling
Source: J Clin Med. 2026 May 29;15(11):4201. doi: 10.3390/jcm15114201 (PMC13258338; doi:10.3390/jcm15114201)

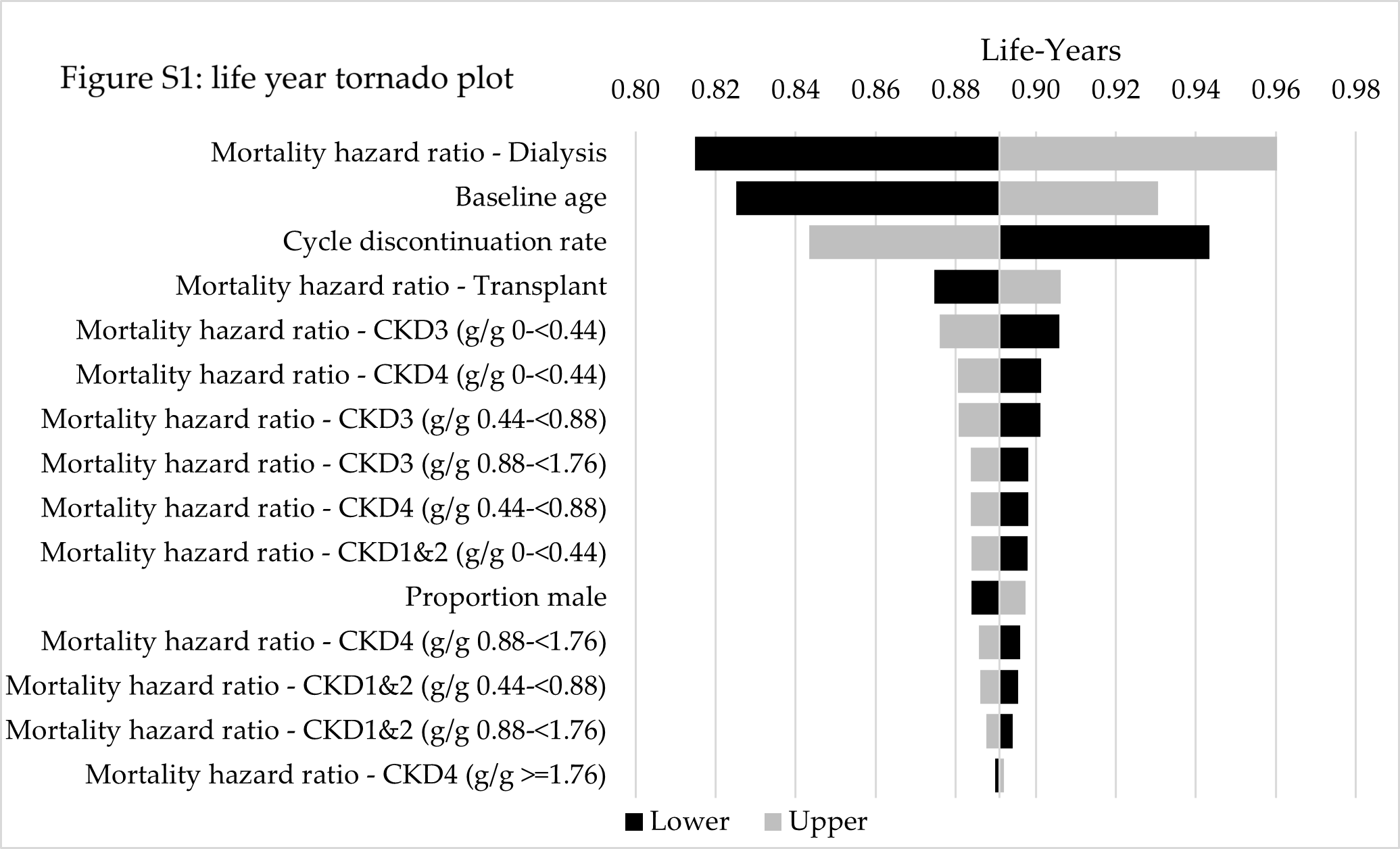

Supplement: Supplementary file 1 [file jcm-15-04201-s001.zip › Figure S1-life year tornado plot.png]

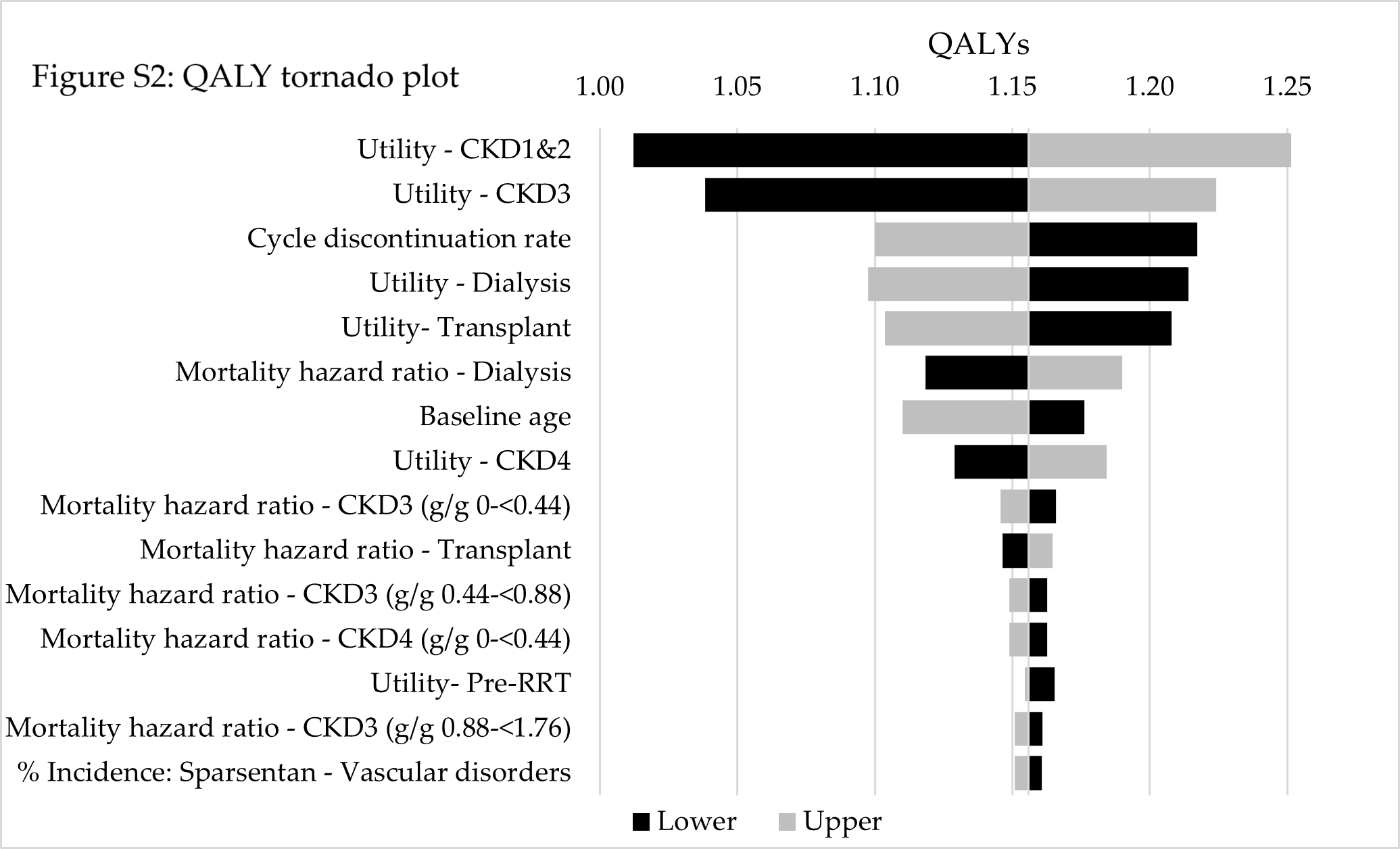

Supplement: Supplementary file 1 [file jcm-15-04201-s001.zip › Figure S2- QALY tornado plot.png]
